# Tilted State Population of Antimicrobial Peptide PGLa Is Coupled to the Transmembrane Potential

**DOI:** 10.1021/acs.jcim.2c00667

**Published:** 2022-10-03

**Authors:** Lukács J. Németh, Tamás A. Martinek, Balázs Jójárt

**Affiliations:** † Institute of Food Engineering, University of Szeged, Mars tér 7, Szeged HU-6724, Hungary; ‡ Department of Medical Chemistry, University of Szeged, Dóm tér 8, Szeged HU-6720, Hungary; § ELKH-SZTE Biomimetic Systems Research Group, Eötvös Loránd Research Network, Szeged H6720, Hungary

## Abstract

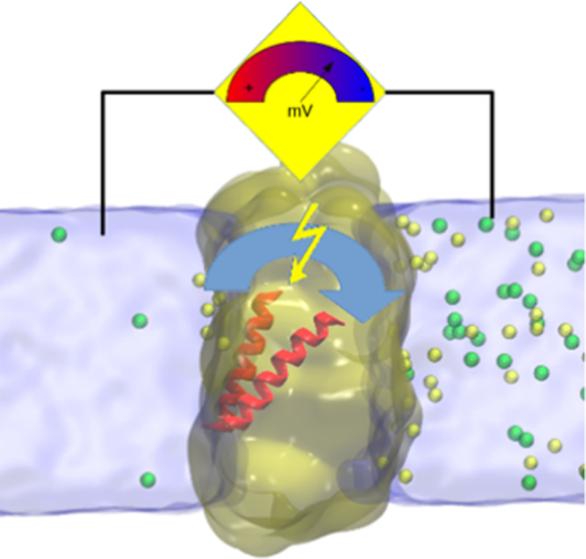

Cationic antimicrobial peptide PGLa gets into close contact with the anionic bacterial cell membrane, facilitating cross-membrane transport phenomena and membrane disruption depending on the concentration. The mechanisms of action are closely associated with the tilted insertion geometry of PGLa. Therefore, we aimed to understand the interaction between the transmembrane potential (TMP) and the orientation of the membrane-bound PGLa helix. Molecular dynamics simulations were performed with TMP, and we found that the PGLa tilt angle relative to the membrane is coupled with the TMP. Elevated TMP increases the population of the tilted state. We observed positive feedback between the tilt angle and the TMP, which occurs due to the electrostatic interaction between the peptidic helix and the Na^+^ cations at the membrane–water interface. These TMP coupled phenomena can contribute to understanding the direct antimicrobial and adjuvant effects of PGLa in combination with regular antibiotics.

## Introduction

Antimicrobial peptides (AMPs) are promising candidates to fight against ever-adapting bacterial pathogens.^1,2^ As host defense peptides, they play a ubiquitous role in the nonadaptive immune system. For most AMPs, the mechanism for action requires binding to the predominantly anionic cell membrane,^3^ which facilitates the binding of cationic AMPs by electronic attraction. The exact mechanism by which binding translates into cell death remains debated. Numerous explanations were put forward, such as crowding, pore formation, membrane thinning, and induced helical propensity, but no single theory has gained sole acceptance so far.^3,4^

These peptides often have an amphiphilic helix structure that requires the negatively charged membrane surface to fold into the bioactive conformation.^5−7^ PGLa is such an example, first isolated from the skin of the frog *Xenopus laevis*. It is a 21-amino-acid peptide with a net charge of +5 that folds into a helix on the membrane surface. Besides its direct antibiotic effect, PGLa acts as an adjuvant administered in combination with small molecule antibiotics.^7^ These experiments concluded that the peptide insertion into the membrane induced hyperpolarization, and the elevated transmembrane potential (TMP) was confirmed in vesicular model systems. Membrane potential also directly influences the uptake of ionic material and the orientation of molecules exhibiting a net dipole moment.^8,9^ Cationic antimicrobial peptides feature a combination of these properties; therefore, the incorporation of TMP into molecular dynamics simulations offers new insight into their dynamic interactions with the membrane.

Membrane potential in molecular dynamics simulations can be generated in three ways: applying an external electric field via direct tensor,^10,11^ introducing net ion imbalance between the extra- and intracellular volume,^12^ and applying salt concentration gradient across the membrane.^13^ The direct tensor method is straightforward, but it can only maintain uniform field strength poorly suited for simulating abrupt changes in dielectric strength, such as protein insertion into the membrane. The net ion imbalance approach produces a significant level of membrane potential. However, the potential is very sensitive to the number and fluctuation of ions. For regular-sized systems, one net charge difference may already generate unrealistic TMP. These effects can be alleviated using the double bilayer salt-gradient method. The salt accumulates at the lipid surface, and Na^+^ has a high concentration at the surface, whereas Cl^–^ at some distance, and thus, the charge separation allows well-tunable TMP.

In this study, we tested a double bilayer membrane compartment system with salt concentration gradients to model TMP and its effects on the membrane insertion behavior of the antimicrobial peptide PGLa.

## Materials and Methods

**Figure 1:**
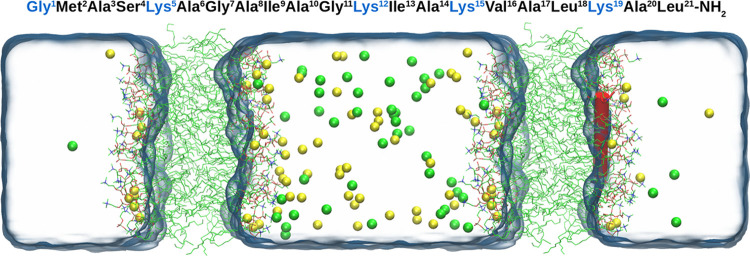
Sequence of PGLa and the double bilayer system simulated with salt gradient (DB.S.P). The water box is represented as a transparent slab. Green and yellow spheres correspond to chloride and sodium ions, respectively. Membranes are depicted as line art, and the peptide PGLa is represented as cartoon in red. Positively charged residues in the sequence are highlighted in blue.

In this study, we simulated five systems (Table 1): DB.S: a double bilayer with 0.4 M NaCl (44 Na^+^ and 44 Cl^–^) present in the intracellular part and counterions (32 Na^+^); NIIMB: a double bilayer with a net ionic imbalance between the water compartments;^12^ DB: a double bilayer with counterions only (32 Na^+^); SB.P: a single bilayer with counterions and the peptide (16 Na^+^ and 5 Cl^–^); DB.S.P: a double bilayer with 0.4 M NaCl present in the central compartment, counterions, and the peptide (76 Na^+^ and 49 Cl^–^) (Figure 1).

**Table 1:** Summary of the Simulated Systems^a^

ID	TMP	PGLa	*N*_b_	*N*_a_	trajectory length (whole/analyzed)
DB.S	yes	no	2	58 378	500/375 ns
NIIMB	yes	no	2	88 296	50/10 ns
DB	no	no	2	58 554	500/375 ns
SB.P	no	yes	1	29 586	500/375 ns
DB.S.P	yes	yes	2	58 687	500/375 ns

a TMP – transmembrane potential; *N*_b_ – number of bilayers; *N*_a_ – number of atoms.

### Preparation of the Systems

For the SB.P simulation, the final frame of our recently published simulation of the membrane-induced folding of PGLa^7^ was used as the initial structure, and the composition of the membrane model was DOPC/DOPG at the ratio of 80:20. All DB systems were created from this structure by the method of duplicating and translating the system in the *z*-direction and modifying the structure obtained. In the case of DB, peptides and their counterions were deleted from the structure. For the DB.S.P system, we substituted the desired number of water molecules with Na^+^ and Cl^–^ ions in the center compartment to achieve the concentration difference, and one PGla was removed from the structure (Figure 1). The DB.S simulation setup was prepared by deleting the peptide and its counterions from the DB.S.P system. For the NIIMB simulation, we applied the structure described in Lai et al.,^14^ and the net ion imbalance was set to +8.

### Molecular Dynamics Parameters

The structures were minimized and heated in two steps, first from 10 to 100 K for 500 ps and subsequently from 100 to 310 K for 1000 ps using the NVT ensemble. After heating, except for NIIMB (see Table 1), 500 ns simulations were performed, and the last 375 ns trajectory part was analyzed. The constant temperature was maintained using Langevin dynamics,^15^ and a Berendsen barostat^16^ was used in the NPT step; electrostatic interactions were calculated with the particle mesh Ewald method,^17−19^ and the cutoff value for nonbonded interactions was set to 10 Å. During the NPT calculations, we applied semi-isotropic pressure scaling, and the surface tension was set to 0 dyn/cm. Molecular dynamics simulations were performed with the AMBER16 program package^20^ employing the CUDA code.^21,22^ Water molecules, the peptide, and the lipids were modeled with TIP3P,^23^ ff14SB,^24^ and lipid14^25^ force field parameters, respectively. For ions, Joung–Cheatham parameters were used.^26,27^ We applied the hydrogen mass repartition method throughout the simulation, allowing a 4 fs time step.^28^

### Analysis of the Molecular Dynamics Trajectories

First, the transmembrane potential and density were calculated using the potential and density modules implemented in GROMACS version 2018.3.^29^ The gmx potential utility sums the partial charges of all atoms in the simulation into *n* slices along the *z* coordinates (*n* was set to 1050 and 2100 for single and double bilayer simulations, respectively). Second, this charge distribution is substituted into the Poisson equation and doubly integrated, yielding the potential across the box perpendicular to the plane of charge summation. All charges in the force field parameters are taken into account in the calculation, including net charges on ions and the partial charges calculated with the RESP method. The cluster analysis was performed with the *kmeans* function implemented in R software.^30^ The free energy profile was calculated using the *hist* module of cpptraj (version 4.25.6)^31^ with a bin width of 0.05 Å at the temperature of 310 K. Free energy values were obtained with the equation *G*_*i*_ = −*k*_B_*T* ln(*N*_*i*_/*N*_Max_), where *k*_B_ is the Boltzmann constant, *T* is the temperature, *N*_*i*_ is the population of bin *i*, and *N*_Max_ is the population of the most populated bin.

## Results and Discussion

**Figure 2:**
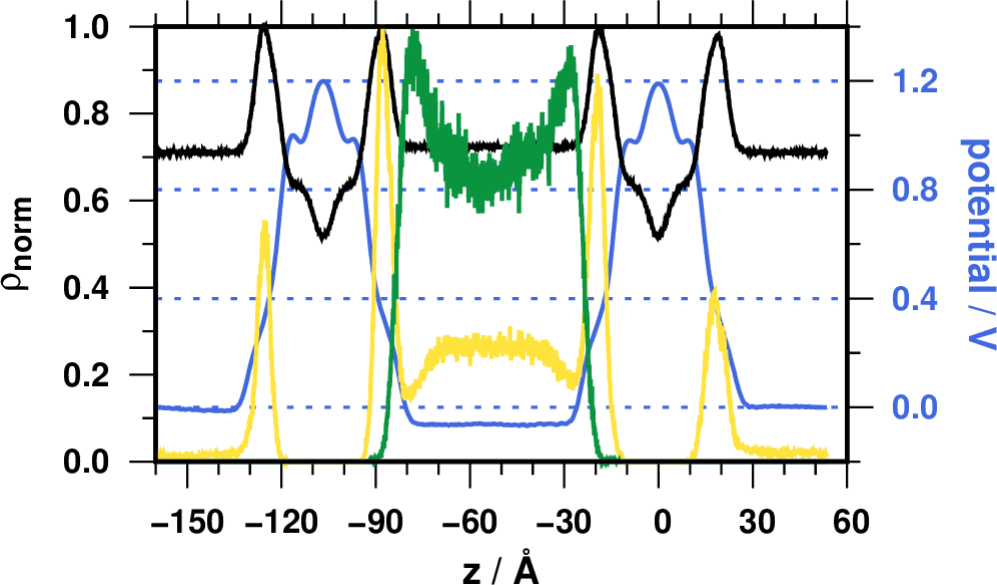
Normalized density (ρ_norm_) profiles (black, system; yellow, Na^+^; green, Cl^–^) and potential curve (blue) for the DB.S simulation.

The TMP was generated with 0.4 M NaCl added to the central compartment (Figure 1), and the excess salt concentration was set based on literature results on the dimyristoyl phosphatidylcholine (DMPC) membrane.^13^ To consider the longer fatty acid chain of oleyl chains, we increased the concentration (0.4 M NaCl) to obtain a TMP in the desired range. For the DB.S simulation, the static membrane potential of −66 ± 28 mV (negative side in the central compartment, values are given as average ± standard deviation) was established and maintained throughout the simulations (Figure 2).

This setup approximates the biologically relevant regime,^32^ and the central compartment mimics the intracellular space. The potential is static in the sense that no diffusion potential is involved. However, there was a considerable fluctuation in the TMP over time due to the dynamics of the ions.

We found that the net TMP does not affect the overall appearance of the potential curve. The potential diagram is dominated by the large positive peak of dipole potential inside the membrane (Figure 2).^33^ The TMP is an order of magnitude lower than the potential barrier observed inside the membrane. We compared our results with the literature findings, where the membrane potential was generated through net charge separation across the membrane.^14^ Strikingly, the charge separation approach (the NIIMB simulation) yields a TMP of 4000 mV in our hands (Figure S1), which has an immediate and strong distortion effect on the membrane such as random peptide and water translocation in accordance with the literature results. Considering that 4000 mV is far from the biologically relevant TMP range, we based our further simulations on the salt-gradient method.

In the absence of excess NaCl in the central compartment (DB, Figure S2), the system displayed a residual net TMP of +4 ± 27 mV. The large standard deviation compared with the small overall magnitude of the potential results from the lower ionic strength of the solution, decreasing the screening effect of the solvated ions and extending the Debye length. Statistically, this small TMP is not different from zero in our case. However, systematic bias has been observed in literature simulations, and it has been attributed to the finite-size effects of the simulation box.^12^ We intended to eliminate any potential unwanted artifact in the control setup. Therefore, the TMP-free simulations with the peptide were performed in a single bilayer setup (SB.P), where periodic boundary conditions ensured zero TMP.

**Figure 3:**
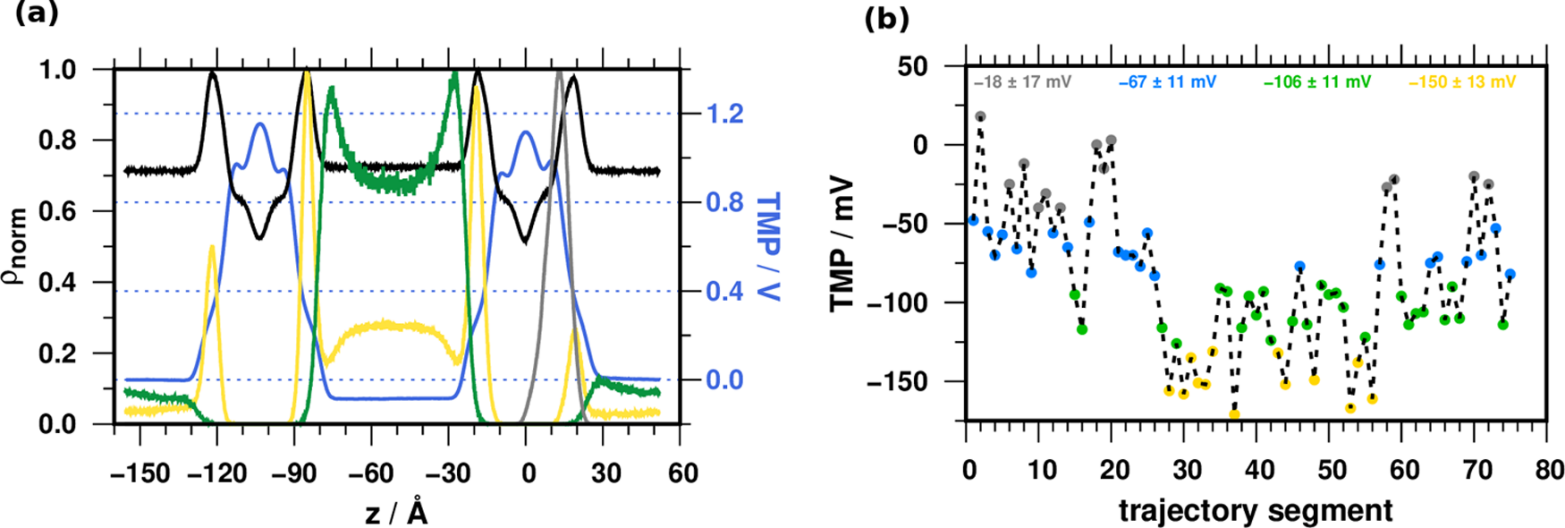
(a) Normalized density (ρ_norm_) profiles (black, system; yellow, Na^+^; green, Cl^–^) and potential curve (blue) for the DB.S.P simulation. (b) TMP for the DB.S.P simulation, calculated from 5 ns slices. Colored points represent the clustering of the individual potential data. The top color-coded row shows the average TMP and standard deviation for each cluster.

Incorporating peptide PGLa into the double bilayer system with excess salt in the central compartment (DB.S.P) enhances the already existing negative TMP to −87 ± 44 mV (Figure 3a).

Slicing up the 375 ns trajectory to 5 ns segments and calculating the potential profile and TMP for each slice reveal that the apparent growth of the standard deviation is due to a hyperpolarization event (Figure 3b). Hyperpolarization of the membrane was associated with AMP insertion in recent experimental studies;^34,35^ therefore, we set out to test how the peptide behavior changes during these events.

DB.S.P and SB.P simulations were started from the same initial peptide structure, where PGLa was parallel with the membrane surface. We investigated the density distribution of the PGLa atoms along the *z*-axis with the membrane center as the reference point. For the DB.S.P simulation, the distribution has a bimodal nature that can be decomposed into two Gaussian functions. The distribution obtained for SB.P simulation also shows a low level of skewing, but the decomposition shows a significantly lower contribution from the component closer to the membrane center (Table S1 and Figure S3).

**Figure 4:**
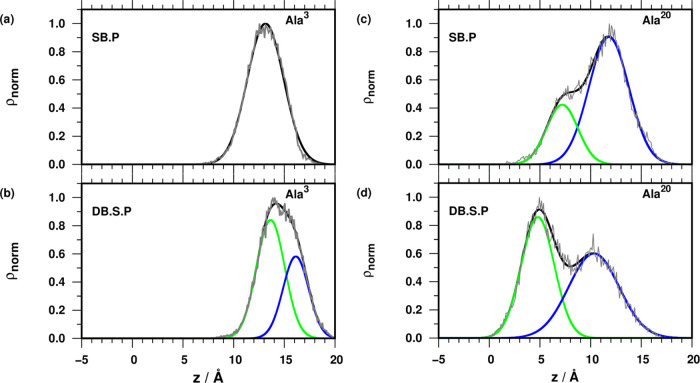
Density profiles of Ala^3^ atoms for SB.P (a) and DB.S.P (b) simulation trajectories and Ala^20^ atoms for SB.P (c) and DB.S.P (d) simulation trajectories (gray, original data, black, fitted Gaussian function; green and blue, unique fitted Gaussian functions). The center of the membrane is at 0 Å, defined as the average position of the terminal methyl groups of the lipids.

Previous experimental studies have implied transition from a surface parallel “S” state to a tilted “T” state in the peptide translocation and membrane disruption process.^36^ We analyzed the peptide distribution at specific residues to test whether the deeper penetration occurs due to uniform immersion or a tilting behavior (Table S1 and Figure 4).

The first two residues at the C-terminus, the last amino acid at the N-terminus, and the NH_2_ capping group were omitted from the analysis due to the higher flexibility of the termini (Figure S4). The normalized densities of the residues Ala^3^ and Ala^20^ show that the C-terminus inserts into the membrane deeper in the presence of TMP. Literature data^5^ and our modeling results strongly suggest that the C-terminus of PGLa dips deeper into the membrane because it is strongly hydrophobic in nature, it lacks charge, and L21 is essential for biological activity. The centers of the Ala^3^ distribution were at 13.1 and 13.6 Å for systems SB.P and DB.S.P, respectively, with an additional peak at 16.1 Å for DB.S.P (Figure 4).

In contrast, a marked difference was found in the distribution of residue Ala^20^ (Figure 4). The control SB.P simulation exhibits two peaks, closer and farther from the bilayer center, and the population ratio is 1:3. In the presence of TMP (DB.S.P), the state inserted deeper becomes more populated compared with SB.P simulation (the ratio of the two populations is roughly equal). An additional effect of the TMP is that both distribution maxima are shifted toward the membrane center by 2.4 and 1.5 Å for the internal and external distribution peaks, respectively (Table S1). Independently of the simulation setup, the overlap between the component Gaussian functions modeling the distribution of Ala^20^ indicates a continuous fluctuation in the position along the *z*-axis. Our findings show that a moderate level of TMP is sufficient to generate immersion of a cationic peptide into the DOPG/DOPC membrane at physiological temperature in a few hundred ns simulation.

Several μs-range MD simulations (5.5–37.2 μs) were performed in the DMPC or DMPC/DMPG membrane for PGLa in the literature,^5^ where approximately one peptide translocation event was obtained every 10 μs. In those simulations, elevated temperature (120–180 °C) was used along with a high peptide/lipid ratio (1:22 or 1:21); furthermore, DMPC and DMPG have lower membrane thickness (*D*_HH_ (DMPC) = 34.4–35.3 Å)^37,38^ relative to DOPC (*D*_HH_ (DOPC) = 36.9–37.1 Å).^39,40^ These conditions facilitated the deeper immersion and, consequently, the translocation. In recent research, replica exchange dynamics was employed without an elevated TMP^41^ to model the PGLa–membrane interactions. This study also found a two-state binding, a membrane-inserted, and a surface-bound form, but the N-terminus segment of the peptide remained unfolded. These simulations applied high-temperature simulation threads to explore low-populated, membrane-inserted geometries. Our results indicate that elevated TMP at biologically relevant temperatures can be a useful approach to sample the phase space of the PGLa–membrane system.

During the SB.P simulation, PGLa has a strong helical preference along the whole sequence, and a fraying of the N-terminus (residues 1–5) was observed (Figure S4). This model agrees with the literature on secondary chemical shifts, NOE, and solid-state line shape data.^42^ As the peptide remained helical throughout our simulations (Figure S4), the deeper insertion of the C-terminus requires tilting inside the membrane. The tilt angle (τ) is the angle between the vector connecting the center of mass of residue 3 and residue 20 and the unit vector in the +*z* direction.^43,44^ The tilt angle distribution was calculated using a 1° bin width (Figure S5). In the absence of TMP (SB.P), the most populated region is at around 90°, corresponding to a parallel orientation with the membrane surface. However, higher angles are accessible for the system, indicating the intrinsic ability of the PGLa helix to tilt into the membrane. In the presence of TMP (DB.S.P), a marked shift toward larger τ values was observed. The most populated angles are located at around 110°, and the ratio of structures with τ > 120° is 8%, which is 40 times higher than that obtained in the SB.P simulation. We observed that charged Lys side chains are in close contact with the phosphate moieties of the membrane even at high tilt angles, snorkeling up from the peptide backbone to the charged lipid layer. The Lys side chain length limits the peptide tilt depth^45,46^ until interaction with mobile ions, and desolvation enables further insertion of the helix and cross-membrane ion transport.^7^ Tilted peptide populations were obtained in previous studies by a synergistic interaction with magainin 2^44,47^ or other PGLa peptides with increased *P*/*L* ratio.^5^ Sampling the slow orientational and the conformational fluctuations is challenging for AMPs.^48^ Our results show that TMP is essential for modeling PGLa’s tilting behavior in accessible simulation time scales.

**Figure 5:**
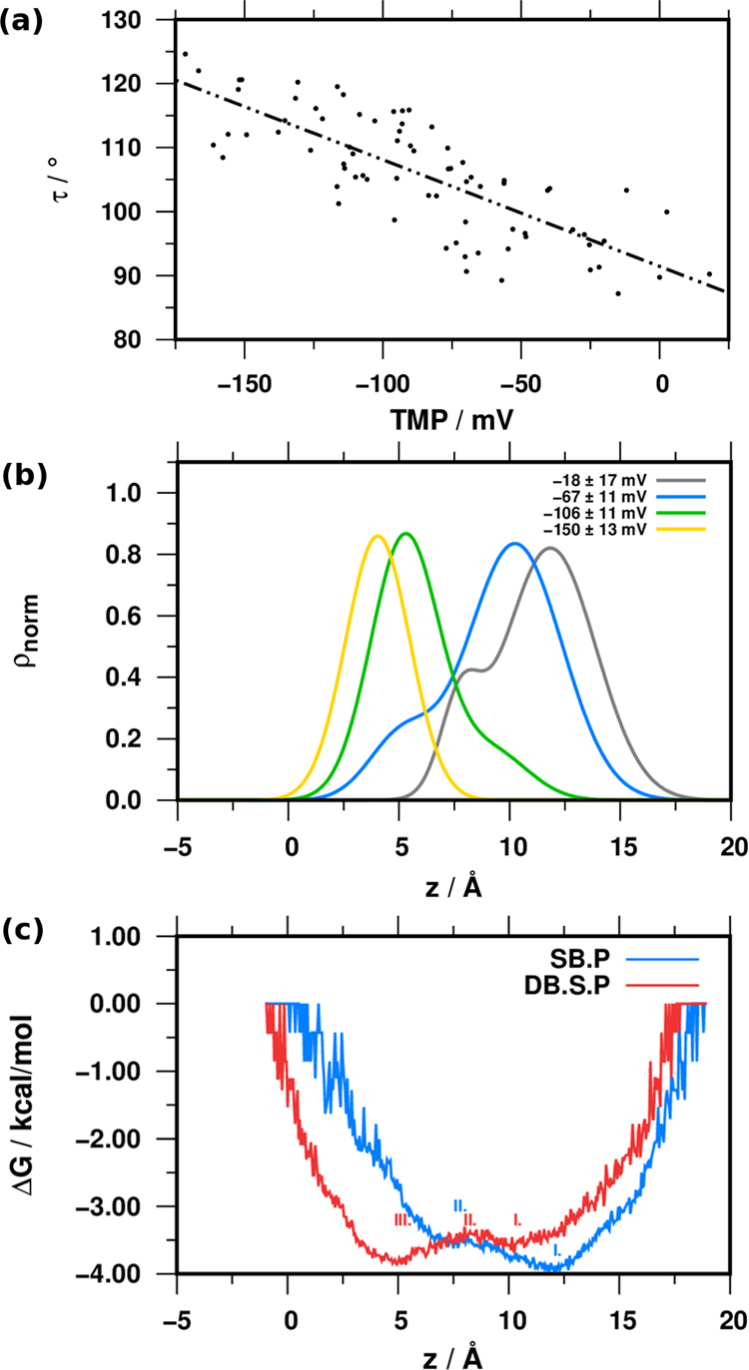
(a) Relationship between the TMP and the tilt angle τ. (b) Distribution of Ala^20^ in the four clusters along the *z*-axis (see Figure 3b). (c) Free energy profiles calculated for the position of Ala^20^ along the *z*-axis. Roman numerals designate local minima and maxima along the PMF curve. For the energy values, see Table S2. Blue and red curves indicate SB.P and DB.S.P simulations, respectively.

Our results show that PGLa tilting is enhanced in the presence of TMP, indicating a correlation between these variables along the simulated trajectories. To test this hypothesis, we divided the trajectory obtained for the DB.S.P simulation (375 ns) into 5 ns windows and calculated the average τ and TMP for each segment (Figure 5a).

The linear regression resulted in an *r*^2^ of 0.6, indicating that the TMP’s overall variance correlates with the tilt angle changes: high tilt angles occur at high negative TMP values (Figure 5a). Considering the lower propensity to tilt with zero initial TMP (SB.P), these results support that TMP facilitates the deeper immersion of PGLa. Plotting the distribution of Ala^20^ for the previously identified TMP clusters further corroborates the presence of the correlation; the deeper insertion of Ala^20^ is associated with the hyperpolarization of the membrane (Figure 5b).

Considering the mechanism that exerts the force on the peptide, we speculate that the gradient of the TMP translates into an electric field strength, which can exert a torque on the peptide helix forcing the helix to pivot. The free energy profile calculated along the *z*-axis position of residue Ala^20^ well reflects the preference for the tilted state relative to the surface orientation (Figure 5c). The potential energy profile experienced by the peptide during immersion into the membrane is shaped by the presence of TMP. Without TMP (SB.P), the lowest energy point is closer to the membrane surface. In the DB.S.P, the lowest energy point is the minimum closer to the center of the membrane, and a potential barrier rises. This energy barrier can be overcome by thermal energy. Thus, it is not high enough to stabilize either position (Table S2).

**Figure 6:**
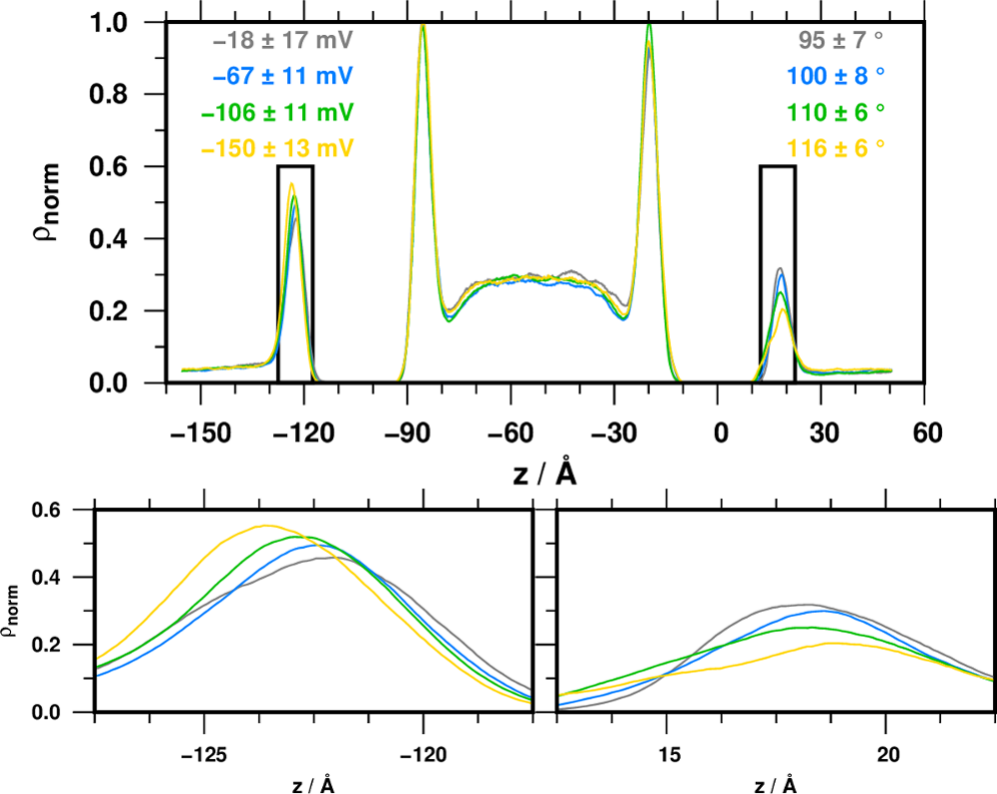
Distribution of Na^+^ along the *z*-coordinate for the four clusters from the DB.S.P simulation. TMP and tilt angle values (average ± standard deviation) are given on the left- and right-hand sides, respectively. The sodium concentration profiles in the membrane leaflets facing outward are magnified on the lower panels.

High tilt angles are associated with higher negative TMP values than those obtained in the control simulation without the peptide (DB.S). Accordingly, the tilted helix itself enhances the hyperpolarization through positive feedback between the tilt angle and the TMP values. We hypothesized that the mechanism is the tilt angle-dependent electrostatic coupling between the peptidic helix and the ion distributions at the membrane–water interface. We extracted the averaged Na^+^ and Cl^–^ density distribution profiles at the tilt angle–TMP combinations of 95 ± 7°, −18 ± 17 mV; 100 ± 8°, −67 ± 11 mV; 110 ± 6°, −106 ± 11 mV; and 116 ± 6°, −150 ± 13 mV (Figure 6).

For high tilt angles, Na^+^ density experiences a marked decrease at the electrical double layer of the peptide side of the membrane. This change is accompanied by a Na^+^ accumulation at the opposite external side and only a minor Cl^–^ concentration profile change. For the low tilt angle state with TMP of −18 mV, we observed an opposite asymmetry in the Na^+^ distribution in the extracellular compartment. The variation of TMP values over the simulation has two principal components. First, a high-frequency oscillation is observed even in the absence of the peptide with an amplitude of 20 mV due to the random fluctuations of ions. Second, a higher amplitude change over a longer time period is associated with the position of the peptide in the membrane, and the amplitude of change is ca. 100 mV.

## Conclusions

In this work, we successfully modeled the TMP via a salt-gradient-induced asymmetric surface potential. This approach afforded an average TMP of −66 mV, which profoundly affected the position of the cationic antimicrobial peptide PGLa relative to the membrane. In the presence of TMP, PGLa tilts into the membrane, which is accompanied by an overall movement of the peptide toward the membrane center. The simulations revealed that PGLa is moving back and forth between two orientation states relative to the membrane surface: parallel and tilted. The free energy of activation between the orientation states is low, which affords a rapid exchange. Beyond the TMP-induced tilting, we observed positive feedback between the instantaneous level of TMP and the tilt angle. This phenomenon can be explained by the tilt angle-dependent electrostatic coupling between PGLa and the Na^+^ ions at the membrane–water interface. This reorganization of the surface-bound Na^+^ ions leads to hyperpolarization.

## Notes

For the simulations, we used the AMBER16 academic license. The packages used were GROMACS, R, and Ambertools18 which are available free of charge (http://www.gromacs.org/, https://www.r-project.org/, and https://ambermd.org/AmberTools.php). Relevant data are included in the article, and input coordinate and parameter files were uploaded as Supporting Information.

## Supporting Information Available

Input coordinate and parameter files; figures (normalized density profiles and potential curves for NIIMB and DB simulations, density profiles of PGLa atoms for trajectories in SB.P and DB.S.P simulations, and residue-level secondary structure propensities of PGLa and distribution of the tilt angles in SB.P and DB.S.P simulations); and tables (fitted parameters of Gaussian functions and free energy values) (PDF)
Input coordinate and parameter files (ZIP)
