# Supplementary material for: Tilted State Population of Antimicrobial Peptide PGLa Is Coupled to the Transmembrane Potential
Source: J Chem Inf Model. 2022 Oct 3;62(20):4963–9. doi: 10.1021/acs.jcim.2c00667 (PMC9597656; doi:10.1021/acs.jcim.2c00667)
Supplement: Supplementary file 1 — ci2c00667_si_001.pdf [file ci2c00667_si_001.pdf]

Supporting Information

The tilted state population of antimicrobial peptide PGLa is coupled to the transmembrane potential

Lukács J. Németh[a], Tamás A. Martinek[b,c,*], Balázs Jójárt[a,#]

[a]Institute of Food Engineering, University of Szeged, Mars tér 7., Szeged HU-6724, Hungary

[b]Department of Medical Chemistry, University of Szeged, Dóm tér 8, Szeged HU-6720, Hungary

[c]ELKH-SZTE Biomimetic Systems Research Group, Eötvös Loránd Research Network, Szeged, H6720, Hungary

Corresponding Authors

[*]Department of Medical Chemistry, University of Szeged, Dóm tér 8, Szeged HU-6720, Hungary, martinek.tamas@med.u-szeged.hu

[#]Institute of Food Engineering, University of Szeged, Mars tér 7., Szeged HU-6724, Hungary

jojartb@mk.u-szeged.hu

Table S1 Center (zc_1 and zc_2) and area (A_1 and A_2) values of Gaussian functions fitted to specific atom (PGLa, Ala^3, Ala^{20}) distributions for SB.P and DB.S.P systems.

	ID	zc_1[Å]	zc_2 [Å]	A_1 [$Å^2$]	A_2 [$Å^2$]
**PGLa**	**SB.P**	12.7	14.6	2.53	1.01
	**DB.S.P**	11.6	14.2	2.57	2.17
**Ala^3**	**SB.P**	13.1	-	-	-
	**DB.S.P**	13.6	16.1	2.92	1.84
**Ala^{20}**	**SB.P**	7.2	11.8	1.64	4.47
	**DB.S.P**	4.8	10.3	3.49	3.74

Table S2 Free energy values (kcal/mol) for orientation states and saddle points on the free energy surface (see Figure 5c).

ID	I.	II.	III.
**SB.P**	-3.91	-3.54	
**DB.S.P**	-3.58	-3.46	-3.79

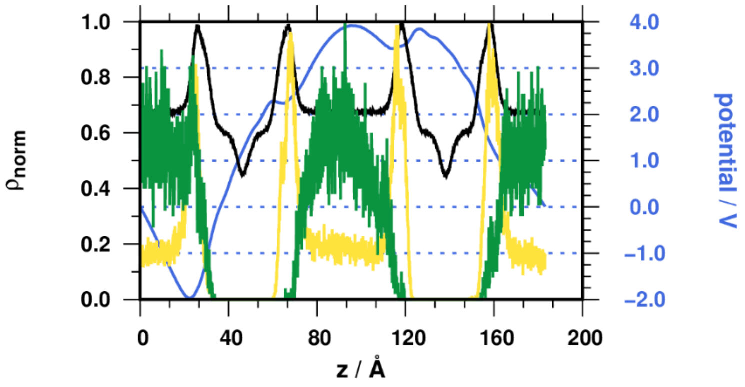

Figure S1 Normalized density (ρ_{norm}) profiles (black - system; yellow - Na^{+}, green - Cl^{-}) and potential curve (blue) for simulation NIIMB.

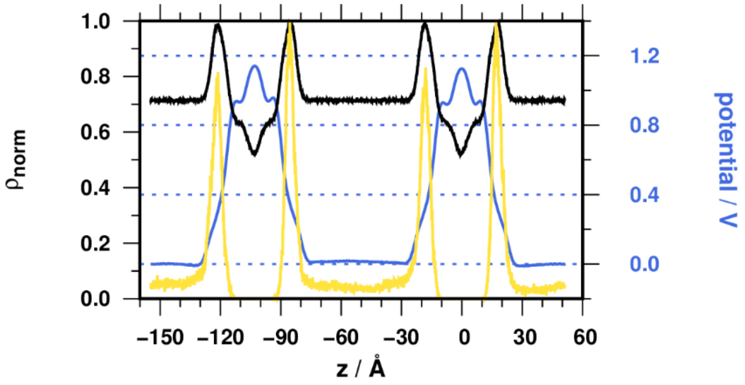

Figure S2 Normalized density (ρ_{norm}) profiles (black - system; yellow - Na^{+}) and potential curve (blue) for simulation DB.

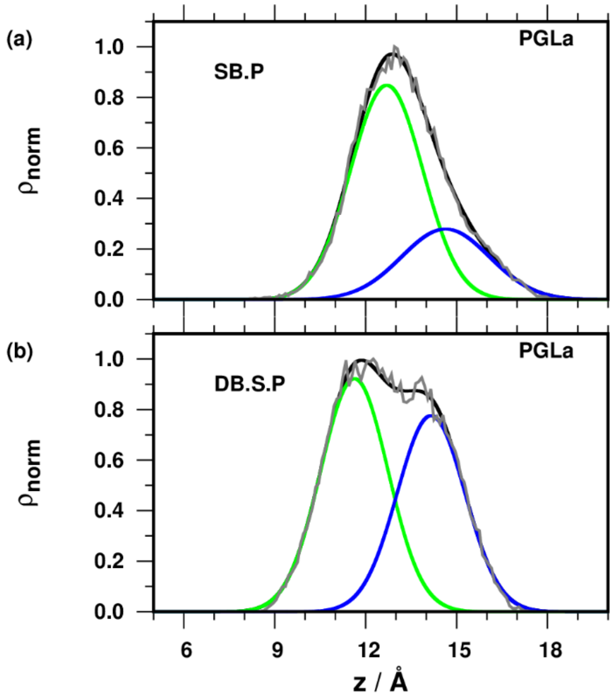

Figure S3 Density profiles of PGLa atoms for trajectories in simulations SB.P (a) and DB.S.P (b) (grey - original data, black - fitted Gaussian function; green and blue - unique fitted Gaussian functions). The center of the membrane is located at 0 Å, defined as the average position of the terminal methyl groups of the lipids.

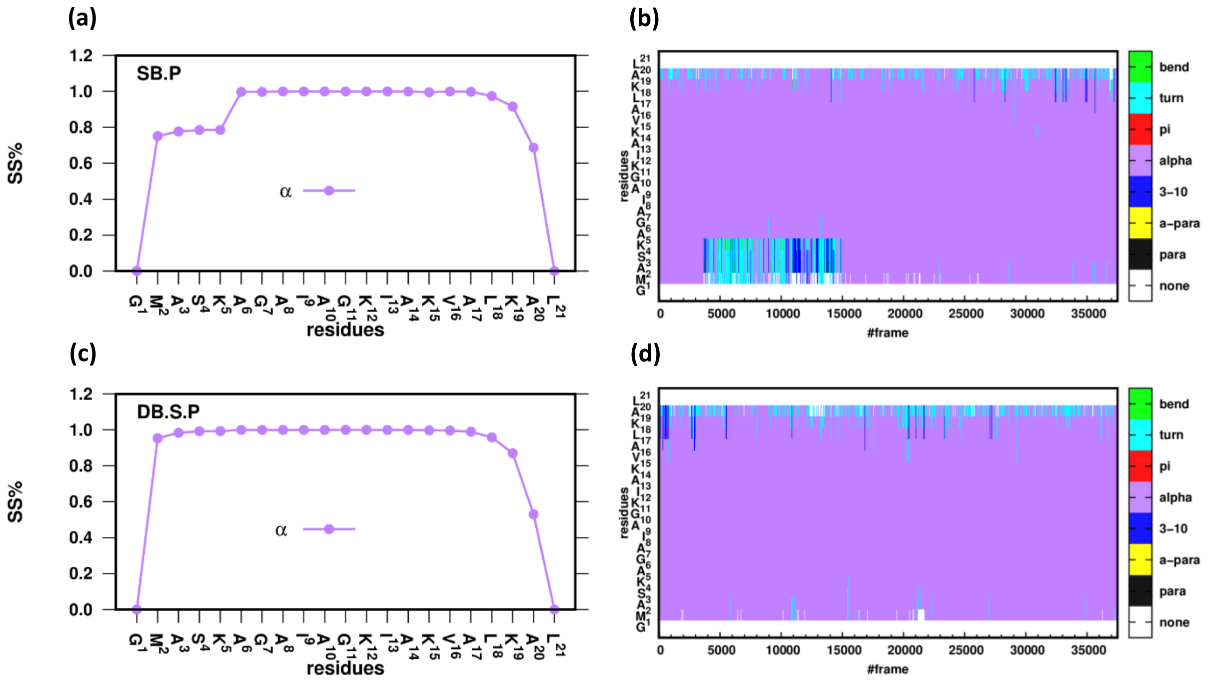

Figure S4 Residue-level secondary structure propensities obtained for PGLa in simulations SB.P (a and b) and DB.S.P (c and d).

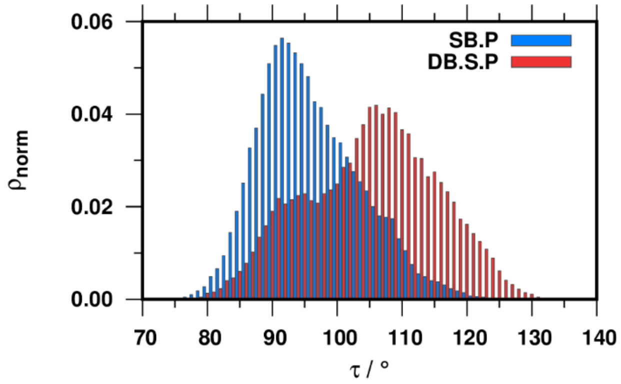

Figure S5 Distribution of the tilt angles (τ) for simulations SB.P and DB.S.P.
